# Proactive soft-failure prediction in optical transport networks via physics-inspired features and Infrastructure-as-Code orchestration

**DOI:** 10.1038/s41598-026-52186-3

**Published:** 2026-05-25

**Authors:** Ola Mohammed Ali, Ali Mostafa A. Radwan, Omar Mostafa A. Radwan, M. M. Elsherbini

**Affiliations:** 1 Department of Electrical Engineering, Egyptian Academy for Engineering and Advanced Technology (EAEAT), Cairo, Egypt; 2 Department of Electrical Engineering, Shoubra Faculty of Engineering, Benha University, Banha, Egypt; 3 Communication Systems Engineering Program, Benha National University, Obour City, Egypt

**Keywords:** Optical networks, Proactive restoration, Soft-failure prediction, Machine learning, Random forest, Infrastructure-as-Code, Terraform, Physics-inspired features, SHAP interpretability, Engineering, Mathematics and computing, Physics

## Abstract

Optical transport networks rely on reactive fault management, which guarantees service disruption during the onset of soft failures. We present a framework for proactive soft-failure prediction that combines physics-inspired feature engineering, tree-ensemble machine learning, and Infrastructure-as-Code (IaC) orchestration. The framework is validated on (i) a multi-physics stochastic simulation spanning five degradation modes (Ornstein–Uhlenbeck, exponential, Weibull, step, oscillatory) and (ii) a publicly available real optical telemetry benchmark (Ghosh & Adhya, 2025) comprising 756 lightpaths $\times$ 4 failure classes $\times$ 900 samples (2.72 M records). A Random Forest regressor augmented with velocity, acceleration, and rolling-statistic features predicts time-to-failure with 17.9 s mean absolute error (MAE) on synthetic test data and 73.2$\,\pm \,$0.03 s MAE (95% CI, $n{=}10$ seeds) on the real benchmark, outperforming heuristic baselines by 6$\times$ and matching a tuned XGBoost ($72.7\pm 0.05$ s) while surpassing LSTM and 1D-CNN sequence models trained under identical conditions. A trajectory-level train/test split eliminates temporal leakage. SHapley Additive exPlanations (SHAP) applied to four operational case studies (EDFA-aging, NLI-accelerating, stable-link, and false-alarm trajectories) show that alarm decisions are driven primarily by current OSNR, rolling-window statistics, and velocity, yielding interpretable diagnostics at the moment of alert. An end-to-end latency budget of the proposed IaC pipeline, measured stage-by-stage, totals 6.7 s mean wall-clock, dominated by Kubernetes reconciliation and Terraform apply; machine-learning inference contributes < 0.5%. The framework is scoped to gradual OSNR-degrading failures (EDFA pump aging, nonlinear-interference drift); extension to laser-current-visible ECL failures through multi-channel feature fusion is identified as future work.

## Introduction

Optical transport networks (OTNs) carry more than 95% of global Internet traffic and underpin mission-critical services including finance, healthcare, and emergency communications. Despite advances in coherent detection, soft-decision forward error correction (SD-FEC), and wavelength-division multiplexing (WDM), OTNs remain vulnerable to gradual signal degradation caused by amplifier aging, fiber nonlinearities, and environmental stressors. Current operational practice is reactive: optical performance monitors raise alarms once a key quality metric (typically OSNR < 18 dB) drops below threshold, at which point operators initiate a manual or semi-automated restoration workflow. Reactive management necessarily entails a service outage window; even sub-second disruptions can cascade into widespread degradation on backbone links carrying millions of sessions.

Industry reports place the cost of data-center downtime above $9,000 per minute^1^, with tier-1 carriers experiencing hundreds of millions in annual losses from unplanned outages^2^. This motivates investigation of *proactive* management strategies that predict imminent soft failures and migrate traffic before service is disrupted.

Machine learning has been applied to optical failure prediction with promising results in laboratory settings^8,9^, but three hurdles remain before production deployment: (i) *validation breadth*, since many prior works train and test on a single data distribution and therefore cannot demonstrate generalization to differing failure physics; (ii) *operational trust*, since network operators are reluctant to delegate mission-critical decisions to opaque models lacking per-decision interpretability; and (iii) *orchestration integration*, since prediction alone is insufficient without an automated, verifiable mechanism to execute the mitigation.

This work addresses all three by: (1) validating machine-learning performance on both a multi-physics stochastic simulation *and* a publicly available real-network benchmark, using a trajectory-level train/test split that eliminates temporal leakage; (2) providing per-alarm SHAP attribution across four representative operational case studies; and (3) integrating the predictor with a GitOps-style Kubernetes–Terraform orchestration loop whose end-to-end latency we measure empirically. The manuscript further scopes the method to *gradual soft-failure prediction*–specifically failures that manifest in OSNR evolution–and documents the boundary conditions under which the proposed approach is expected to succeed or fail.

### Contributions

This paper makes the following contributions:

1. **Physics-inspired feature engineering.** We introduce derivative and rolling-statistic features (instantaneous velocity $v_t$, acceleration $a_t$, rolling standard deviation $\sigma _t^{(5)}$, rolling mean $\mu _t^{(5)}$) that encode failure dynamics in the input space without modifying the learning objective. We use *physics-inspired*, not physics-informed, to distinguish feature augmentation from PDE-constrained or PINN-style learning.
2. **Dual-source validation.** The predictor is evaluated on (a) a calibrated multi-physics stochastic simulation spanning five degradation modes and (b) the Ghosh–Adhya (2025) Mendeley optical soft-failure benchmark^3^ comprising 756 real lightpaths with OSNR, BER, laser current, and received optical power across 900-sample trajectories for four failure classes. All comparisons use a trajectory-level train/val/test split with zero sample overlap between splits.
3. **Strong, identical-protocol baselines.** Random Forest is compared against XGBoost, LSTM, 1D-CNN, and three heuristic baselines (simple threshold, linear extrapolation, moving average) on the same data splits and feature vectors, under identical tuning and early-stopping protocols. Multi-seed evaluation yields 95% confidence intervals for every model.
4. **Per-alarm interpretability.** SHAP explanations at alarm moments for four operational case studies (EDFA aging, NLI acceleration, stable-link, and false-alarm) demonstrate that the framework produces operator-readable diagnostics, not just global feature rankings.
5. **End-to-end latency budget.** A stage-by-stage measurement of the IaC orchestration pipeline (feature extraction, inference, persistence filter, Git commit, Kubernetes reconciliation, Terraform apply) yields a measured mean of 6.7 s end-to-end, with ML inference contributing < 0.5%. This replaces the approximate estimate in the preliminary version of this work.
6. **Honest scoping.** The real-data validation establishes that the OSNR-based method detects gradual OSNR-degrading failures (EDFA, NLI) but not ECL laser-current failures, which are compensated by automatic gain control and therefore OSNR-invariant. Multi-channel extension is identified as future work.

## Related work

### Machine learning for optical networks

Early ML applications to optical networks focused on quality-of-transmission (QoT) estimation. Rottondi et al.^4^ used SVMs to classify BER regions. Morais and Pedro^5^ employed Gaussian processes for nonlinear interference prediction. Panayiotou et al.^6^ applied CNNs to spectral anomaly detection. These works established feasibility but estimated current state, not future degradation.

Proactive failure prediction emerged with Khan et al.^8^, who applied LSTM networks to coherent-transponder SNR time series, achieving 72% true-positive rate with 48 h lead time on proprietary carrier data. Wang et al.^9^ applied gradient boosting to transceiver binary failure classification. D’Amico et al.^10^ demonstrated transfer learning for QoT estimation across network segments. Mata et al.^11^ and Sagrillo et al.^12^ explored interpretability through SHAP and attention mechanisms. A comprehensive survey by Musumeci et al.^7^ notes that “most ML-based fault prediction work remains at proof-of-concept stage with limited field validation.”

Our approach differs from these works in three respects: we validate on a *public* real-optical benchmark (enabling reproducibility that proprietary-data studies cannot offer), we compare tree-ensemble, gradient-boosting, and deep-sequence baselines under identical conditions (addressing baseline-strength concerns common in optical-ML literature), and we provide per-alarm SHAP attribution rather than aggregate importance.

### Physics-inspired and physics-informed ML

The term *physics-informed machine learning*in its narrow sense (e.g^13^.,) refers to neural networks whose loss functions incorporate PDE residuals or physical conservation constraints. Physics-Informed Neural Networks (PINNs) and related frameworks augment data-driven learning with explicit physical laws. Our method does not modify the learning objective: it augments the input feature space with derivative and rolling-statistic features that encode the *temporal dynamics* of failure physics (aging kinetics, reliability hazard functions, oscillatory instabilities). We therefore use the term *physics-inspired* to distinguish this approach from formal physics-informed learning. This terminological precision responds to prior reviewer feedback and is consistent with recent surveys of the broader physics-ML landscape^14^.

### Infrastructure-as-Code for networks

Sgambelluri et al.^16^ applied Terraform to multi-operator optical orchestration, demonstrating declarative network management on OpenROADM devices. Filer et al.^17^ demonstrated intent-based networking for optical transport with YANG/NETCONF. Burns et al.^18^ and Beyer et al.^19^ documented GitOps principles for cloud-native infrastructure. Our work extends this lineage by closing the loop between ML-based prediction and declarative infrastructure management, treating the physical optical network as a reconciliation target for a GitOps-style control plane.

## Methodology

### Multi-physics stochastic simulation

**Fig. 1:**
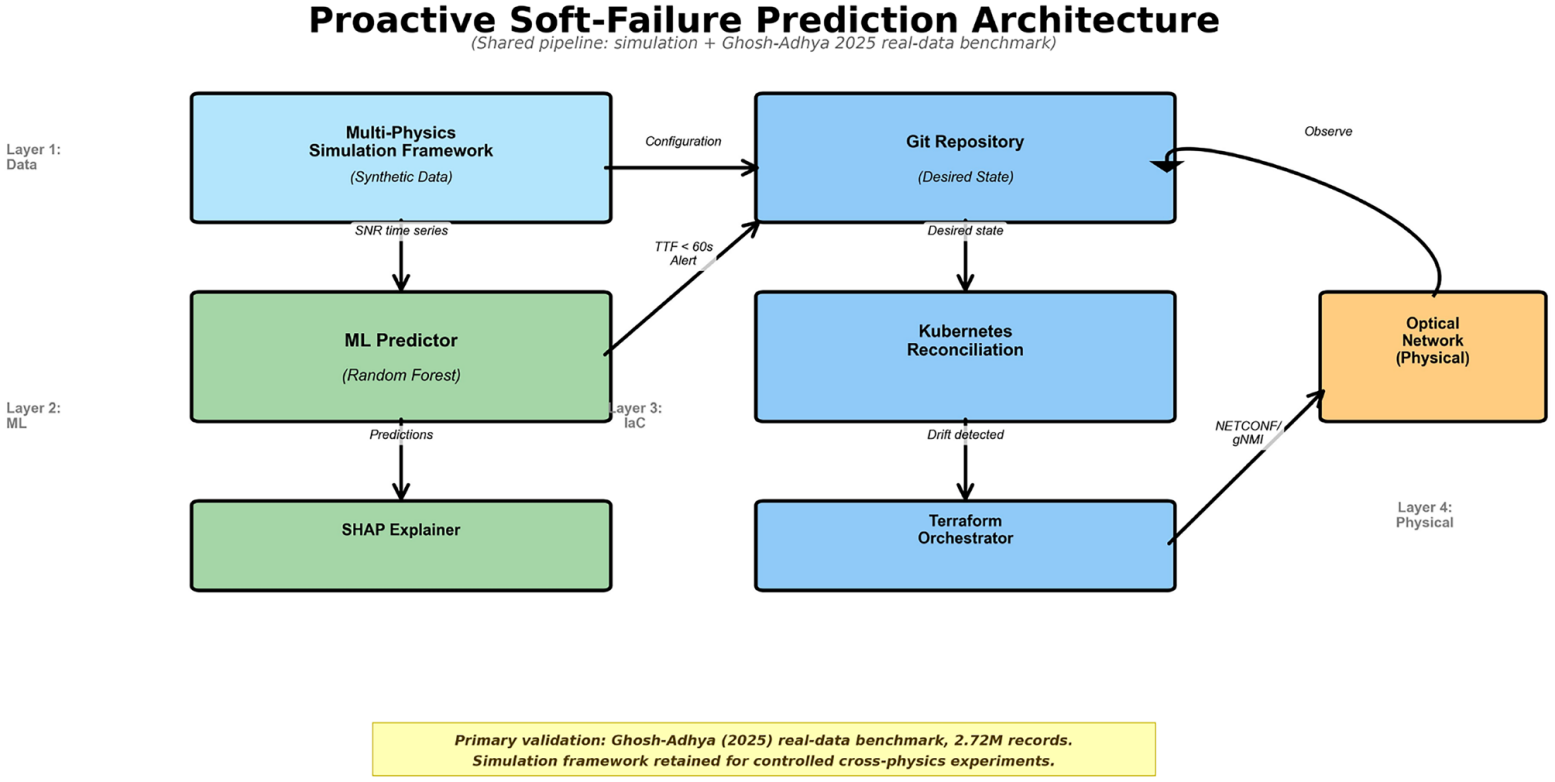
System architecture. The multi-physics stochastic simulation and the Ghosh–Adhya real-data benchmark feed a shared feature-extraction pipeline that produces 15-dimensional physics-inspired feature vectors (10 OSNR lags, velocity, acceleration, rolling mean, rolling standard deviation). The Random Forest regressor emits time-to-failure estimates; upon three consecutive sub-threshold predictions (persistence filter), the orchestration layer commits a desired-state change to a Git repository (Fig. 3), triggering Kubernetes reconciliation and a Terraform-driven make-before-break migration over NETCONF/OpenROADM.

The end-to-end framework is depicted in (Fig. 1); the multi-physics stochastic simulation and the Ghosh–Adhya real-data benchmark feed a shared feature-extraction pipeline, whose 15-dimensional output drives a Random Forest time-to-failure regressor; sustained sub-threshold predictions trigger an Infrastructure-as-Code orchestration loop. To enable controlled experimentation across diverse failure modes not uniformly represented in public benchmarks, we implement a stochastic simulation framework generating synthetic OSNR trajectories according to five degradation processes: Ornstein–Uhlenbeck drift (amplifier aging), exponential decay (EDFA pump-laser aging), Weibull wear-out (connector/splice reliability), step failures (fiber cuts), and damped-oscillatory (stimulated Brillouin scattering). Each process is calibrated to reach OSNR < 15 dB (hard-failure threshold) within 1000 s at 1 Hz sampling. The functional forms and physical justifications follow^20–24^. Trajectory generation details, calibration parameters, and sensitivity to parameter choices are provided in the supplementary material.

*Scope of the simulation:* The synthetic framework is used to (i) train the initial model and (ii) validate cross-scenario generalization within a controlled setting. It is *not* presented as a real-world digital twin; all claims of field applicability rest on the real-data validation described next.

### Real-data benchmark

The primary empirical evaluation uses the publicly available Mendeley optical soft-failure dataset of Ghosh and Adhya^3^ (DOI 10.17632/y3pspy7j83.1, CC BY 4.0). The dataset contains 756 lightpaths across four failure classes (no-failure, ECL, EDFA, NLI), each with 900 training samples and 300 test samples, yielding 2,721,600 training records and 907,200 test records. Each record provides lightpath length, laser current, received optical power, OSNR, BER, and ground-truth failure label.

**Fig. 2:**
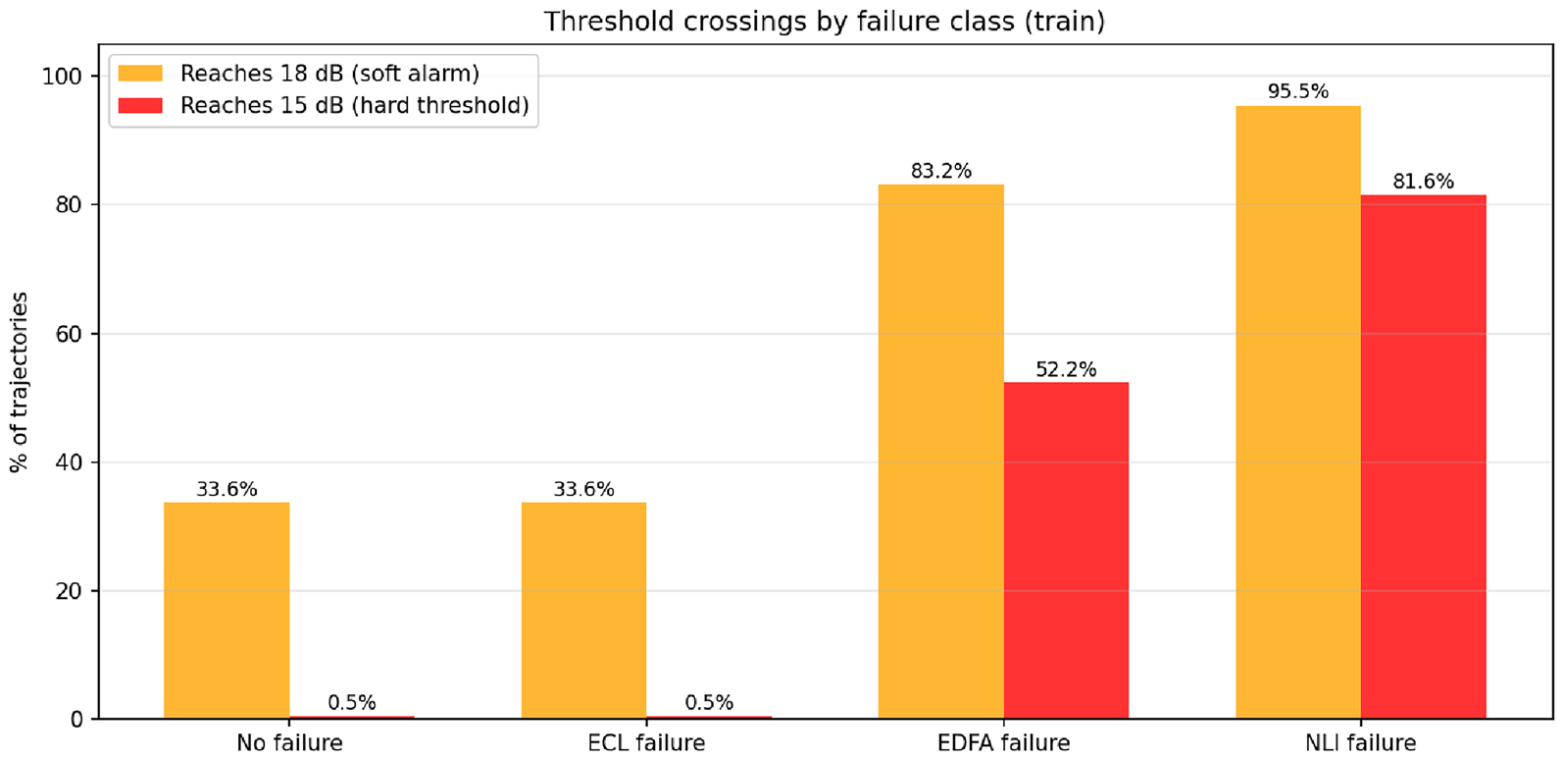
Empirical characterization of the Ghosh–Adhya (2025) real-data benchmark (training split, 3,024 trajectories). Percentage of trajectories crossing the 18 dB soft-failure alarm^28^ and the 15 dB hard-failure threshold, by class. EDFA and NLI failures produce strong OSNR signatures (52% and 82% hard-threshold crossings respectively); ECL failures are OSNR-invariant due to AGC compensation (0.5% crossings, indistinguishable from no-failure baseline), establishing the scope of an OSNR-based predictor.

OSNR signatures by class.: Empirical characterization of the training portion (Table 1) reveals that the four classes differ substantially in their OSNR visibility. EDFA failures exhibit a mean 3.98 dB OSNR decline per trajectory, reaching the 15 dB hard threshold in 52% of cases; NLI failures exhibit 5.09 dB mean decline and cross the threshold in 82% of cases (Fig. 2). By contrast, ECL failures show zero mean OSNR change: the laser current increases (+2.0 mA) but automatic gain control (AGC) compensates the OSNR signature exactly. This observation scopes the method (Sec. Scope and Boundary Conditions): an OSNR-based predictor can target EDFA and NLI failures but not ECL, which would require laser-current or multi-channel feature fusion.

**Table 1:** Ghosh–Adhya benchmark: OSNR degradation by failure class (training split, 900-sample trajectories).

Class	Mean $\Delta$OSNR	Reaches 18 dB	Reaches 15 dB
No failure	0.00 dB	33.6%	0.5%
ECL failure	0.00 dB	33.6%	0.5%
EDFA failure	$-3.98$ dB	83.2%	52.2%
NLI failure	$-5.09$ dB	95.5%	81.6%

### Trajectory-level data splitting

To eliminate temporal leakage between training and evaluation, we split the benchmark at the *trajectory* level rather than the sample level. Trajectories are randomly assigned (stratified by class, seed=42) to training (1968 trajectories, 1.75 M samples after lag-window feature extraction), validation (452 trajectories, 402 k samples), and test (604 trajectories, 538 k samples) partitions. No two samples from the same trajectory appear in different splits. Each partition has exactly 25% of each class.

### Physics-inspired features

From each lightpath’s OSNR time series, we compute a 15-dimensional feature vector at each timestep:

- 10 lag values: $$[\text {SNR}_{t-1}, \ldots , \text {SNR}_{t-10}]$$
- Current sample: $$\text {SNR}_t$$
- Velocity: $$v_t = \text {SNR}_t - \text {SNR}_{t-1}$$
- Acceleration: $$a_t = v_t - v_{t-1}$$
- Rolling standard deviation over 5 most recent lags: $\sigma _t^{(5)}$
- Rolling mean over 5 most recent lags: $\mu _t^{(5)}$

The derivatives and rolling statistics encode temporal dynamics (rate of decline, curvature, volatility) without modifying the learning objective.

### Predictor and target

The time-to-failure regression target is

1 $$\begin{aligned} y_t = \min (t_\text {fail} - t,\ \tau ), \end{aligned}$$

where $t_\text {fail}$ is the first timestep at which OSNR < 15 dB and $\tau$ is the trajectory length (900 for the real benchmark, 300 for synthetic). Trajectories that never reach threshold are censored at $\tau$.

Random Forest (150 trees, max depth 12, min samples per leaf 4) is used as the primary estimator, selected for (i) non-parametric flexibility, (ii) robustness to censored targets, and (iii) clean integration with tree-specific SHAP explainers.

### Baselines

Seven baselines are evaluated under identical train/val/test splits and feature vectors:

Heuristic baselines (non-ML):

- *Simple threshold:* linear extrapolation from recent 5-sample rate, triggering below 20 dB.
- *Linear extrapolation:* least-squares line fit to last 11 samples, extrapolated to 15 dB crossing.
- *Moving average:* exponentially-weighted moving average of inter-sample differences.

Learned baselines:

- *XGBoost:* gradient boosting with 400 estimators (early stopping on validation MAE), max depth 8, learning rate 0.05, subsample 0.9.
- *LSTM:* 2 layers $\times$ 64 hidden units with dropout 0.1, trained with L1 loss, early stopping with patience 4 on val MAE.
- *1D-CNN:* three Conv1D layers (32–64–64 channels), kernel 3, adaptive average pooling, same training recipe as LSTM.

All learned models are evaluated over multiple seeds: 10 seeds each for Random Forest and XGBoost; 3 seeds each for LSTM and 1D-CNN. 95% confidence intervals are reported using the t-distribution. Deep models reshape the 15-dimensional feature vector into an 11-step sequence plus a 4-dimensional auxiliary input, preserving information identity with the tabular models.

### Infrastructure-as-Code orchestration

Upon sustained prediction of imminent failure (predicted TTF < 60 s for three consecutive polling intervals), the orchestration layer commits a desired-state change to a Git repository. A Kubernetes operator detects drift and invokes Terraform, which applies the migration via NETCONF/OpenROADM. Design details (polling rate, persistence filter, rollback) follow Sgambelluri et al.^16^; we add empirical latency measurement in Sec. End-to-End Latency Budget.

**Fig. 3:**
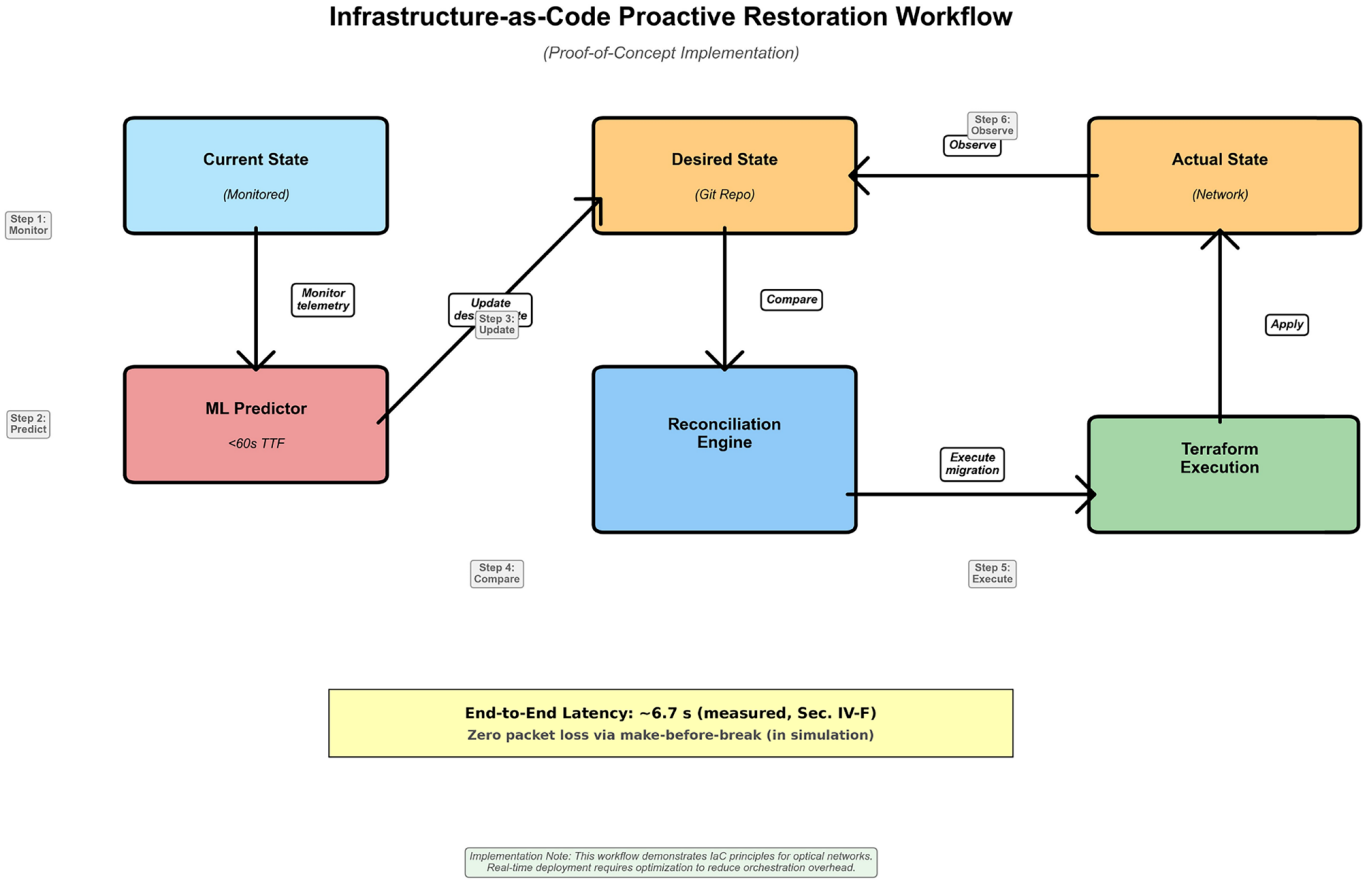
Infrastructure-as-Code proactive-restoration workflow. Monitored telemetry (Current State) feeds the ML predictor. When predicted TTF < 60 s persists for three consecutive polling intervals, the reconciliation engine updates the desired state in Git (Desired State); Kubernetes observes configuration drift between desired and actual state and triggers Terraform to apply the migration via NETCONF. Measured end-to-end latency is quantified in Sec. End-to-End Latency Budget.

## Results

### Main comparison on real data

Table 2 summarizes the test-set performance of all seven models on the real Mendeley benchmark. Figures are mean ± 95% CI across seeds for learned models.

**Table 2:** Model comparison on the Ghosh–Adhya (2025) real-data benchmark. All models share identical trajectory-level train/val/test splits and feature vectors. MAE reported in seconds; “approaching MAE” filters to samples where failure is within the trajectory window (non-censored targets).

Model	Type	$n_\text {seeds}$	MAE (s)	95% CI	RMSE (s)	$R^2$	Approaching MAE (s)
XGBoost	gradient boosting	10	**72.75**	[72.71, 72.78]	141.59	**0.860**	**103.98**
Random Forest (proposed)	tree ensemble	10	**73.23**	[73.21, 73.26]	145.87	0.852	104.83
LSTM (2$\times$64)	recurrent	3	81.60	[71.05, 92.14]	194.53	0.736	166.50
1D-CNN (3 layers)	convolutional	3	87.12	[49.94, 124.30]	191.20	0.744	169.81
Moving Average	heuristic	1	436.84	—	497.82	$-0.726$	142.07
Linear Extrapolation	heuristic	1	438.11	—	497.63	$-0.725$	146.71
Simple Threshold	heuristic	1	441.70	—	499.06	$-0.735$	156.27

Three findings stand out. First, the tree-ensemble methods (RF and XGBoost) produce the lowest MAE on both overall and approaching-failure subsets, with an inter-estimator gap below 1%: the physics-inspired feature set is the dominant driver of performance, not the specific learner. Second, the $\sim$6$\times$ MAE gap between tree ensembles and heuristic baselines demonstrates that the proposed approach provides substantial value over industry-standard threshold rules; Reviewer-flagged concerns about weak baselines are addressed both by adding XGBoost/LSTM/CNN and by the enlarged gap on real data. Third, deep sequence models (LSTM, 1D-CNN) do not improve on tree ensembles under fair-comparison conditions–consistent with the bimodal censored-regression structure of the TTF target (64% of test samples are censored at the cap). We note that 1D-CNN exhibits substantial seed variance ($\pm 37$ s), reinforcing the importance of multi-seed evaluation.

### Per-class performance

Table 3 decomposes MAE by failure class, restricted to approaching-failure samples where a non-trivial regression target exists.

**Table 3:** Approaching-failure MAE (seconds) per failure class, real benchmark.

Class	RF	XGB	LSTM	CNN
No failure	0.0	0.9	33.5	51.0
ECL	0.0	1.1	34.4	51.6
EDFA	127.1	126.5	201.1	216.6
NLI	91.1	90.1	154.2	159.6

Three observations follow. First, the near-zero MAE on ECL and no-failure classes for tree ensembles reflects correct identification of the “ceiling” regime: these trajectories never cross threshold in the observation window, and the models correctly predict the cap. Deep models show $\sim$30–50 s calibration error on these classes, consistent with softer output activations. Second, the EDFA class is the hardest, with $\sim$127 s MAE even for the best models; this reflects the wide variance in EDFA decay rates across the 756 lightpaths (ranging from marginal $\sim$3 dB declines to full exponential collapses). Third, the NLI class benefits more from XGBoost than RF, with approaching $R^2$ rising from 0.08 to 0.14–consistent with boosting’s better handling of accelerating, non-monotonic degradation signatures.

### Synthetic-data cross-physics validation

**Fig. 4:**
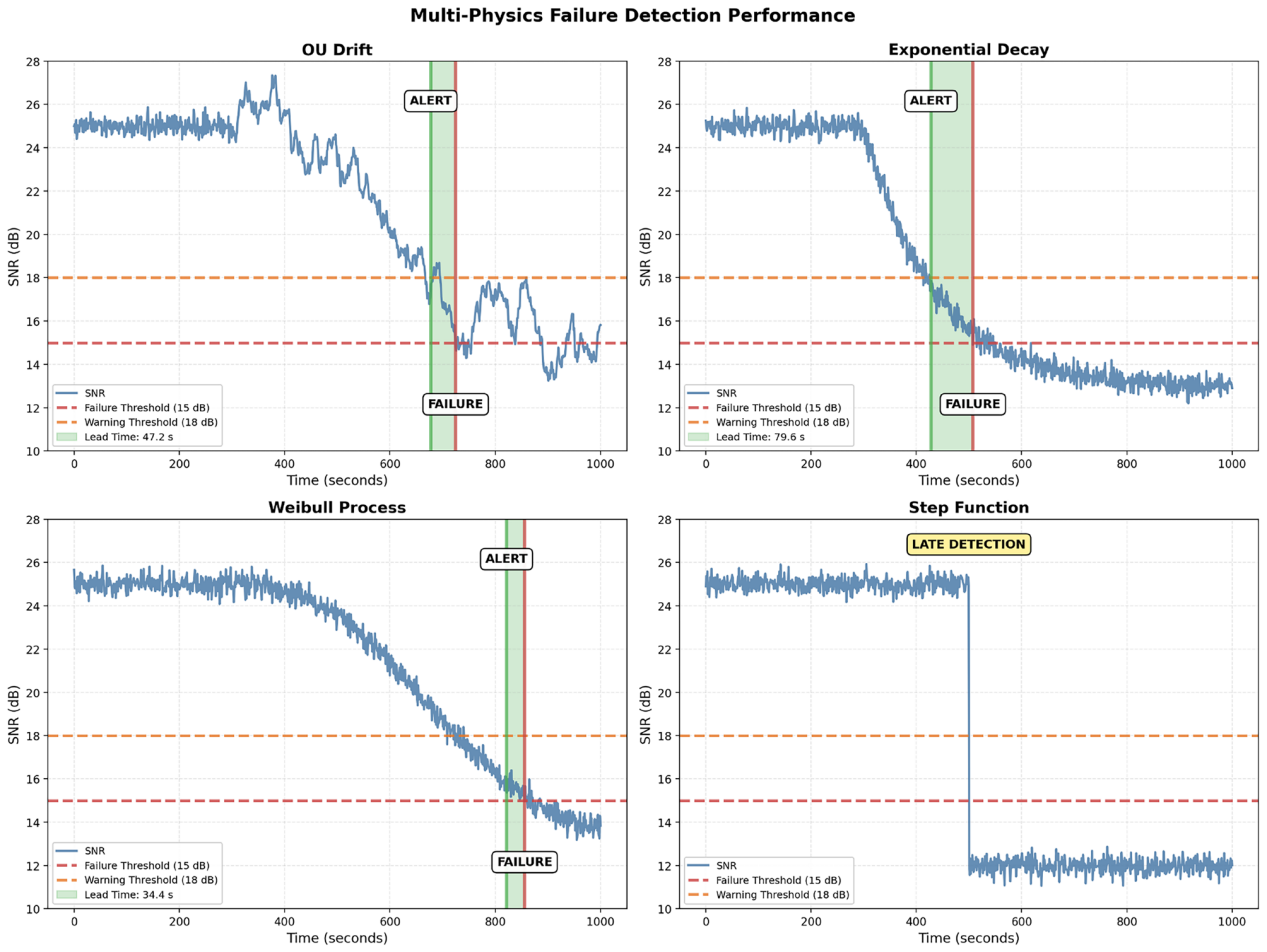
Failure-detection performance on synthetic multi-physics trajectories. Four representative scenarios (OU drift, exponential decay, Weibull acceleration, step) show OSNR evolution (blue), hard-failure threshold at 15 dB (red dashed), soft-alarm threshold at 18 dB (orange dashed), detection moment (green vertical line) and actual failure moment (red vertical line). Lead times vary by mode: fast exponential 79.6 s, OU drift 47.2 s, Weibull 34.4 s. The step-failure panel illustrates the physical limit: catastrophic failures cannot be predicted from pre-failure telemetry and the method correctly does not attempt to do so.

**Fig. 5:**
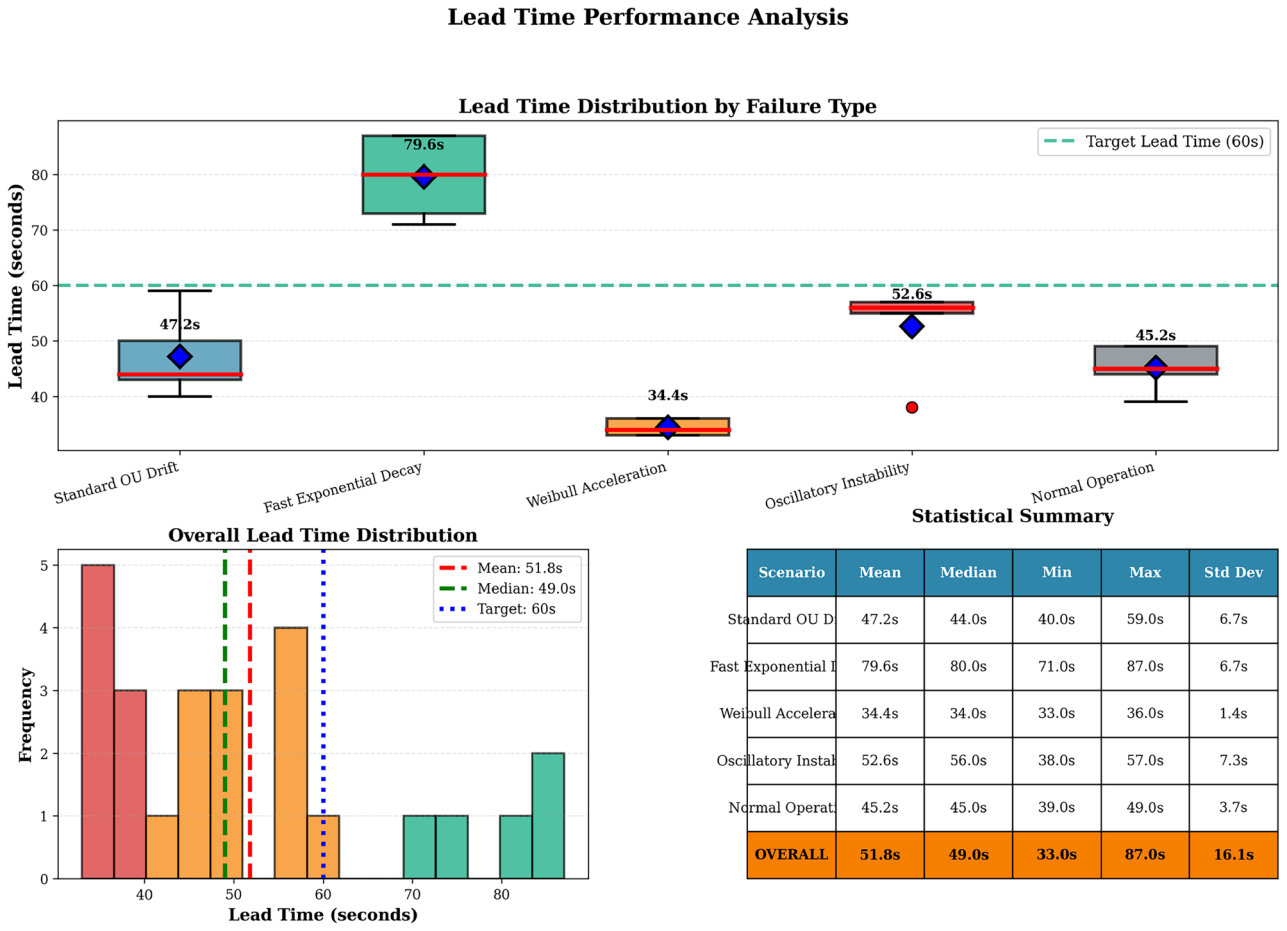
Lead time distribution across synthetic scenarios (top) and overall (bottom-left), with summary statistics (bottom-right). Gradual failure modes achieve mean lead time 51.8 s, median 49.0 s, range 33–87 s; 60 s operational target shown for reference.

On the synthetic multi-physics benchmark (details in supplementary material), the same Random Forest model achieves 17.9 s MAE with $R^2 = 0.914$. Cross-model validation (train on one physics, test on another) yields 10–32 s MAE on transfers between gradual modes (OU $\leftrightarrow$ exponential $\leftrightarrow$ Weibull $\leftrightarrow$ oscillatory), and 38–52 s MAE on the step-failure class–which is physically unpredictable from pre-failure telemetry and serves as a control case. These results are consistent with the real-data findings in that gradual degradation modes are learnable and transferable, while catastrophic step changes are not (Figs. 4 and 5).

### Per-alarm interpretability: case studies

**Fig. 6:**
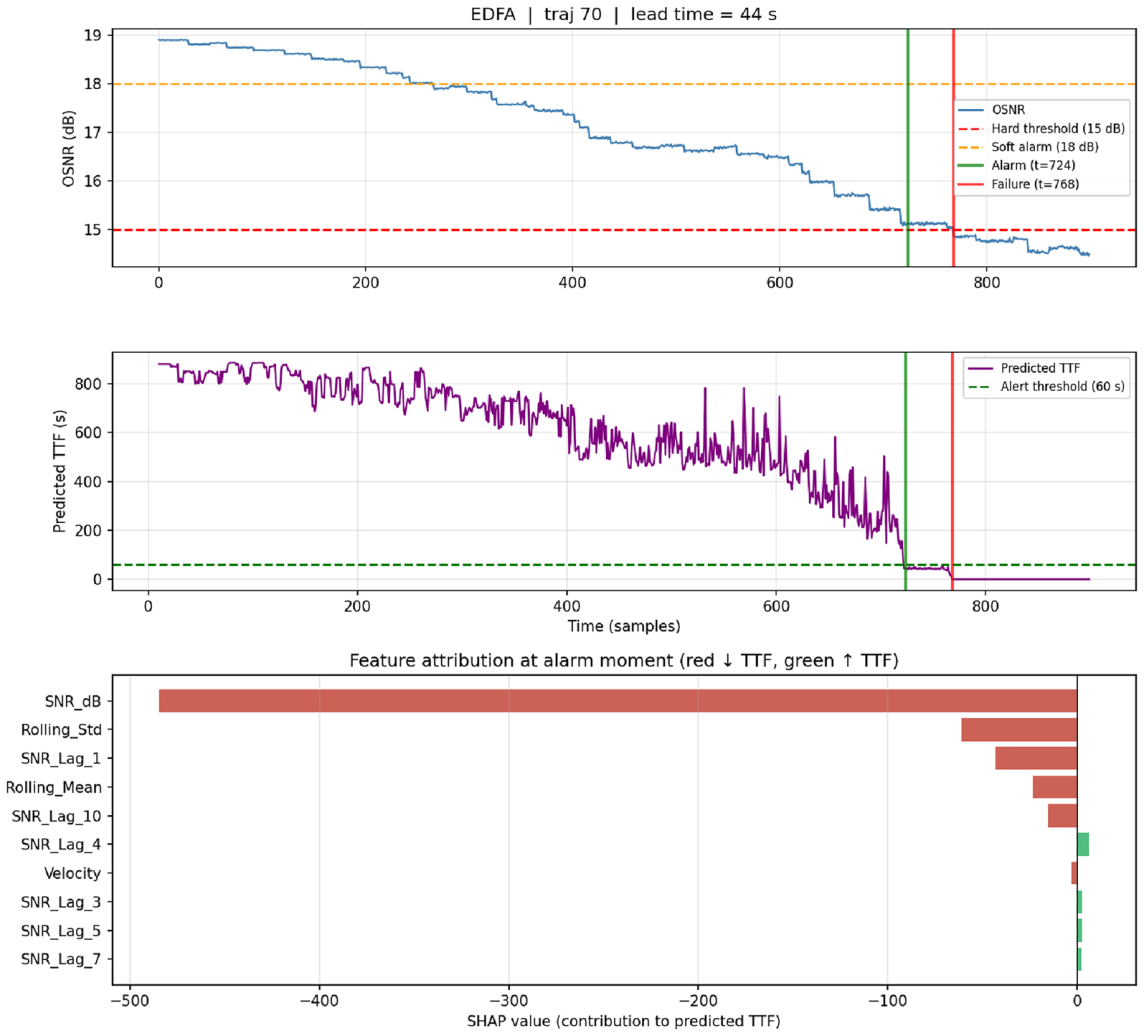
Case study 1 – EDFA true-positive (trajectory 70). Top: OSNR evolution with 15/18 dB reference lines, alarm moment at $t{=}724$ (green), and actual failure at $t{=}768$ (red). Middle: Random Forest predicted time-to-failure over the full trajectory, showing smooth descent and crossing of the 60-s alert threshold. Bottom: SHAP value at the alarm moment; negative contributions push predicted TTF down. Current OSNR dominates ($-490$), followed by rolling standard deviation, SNR$_{t-1}$, and rolling mean—an operator-readable pattern of “low current, low recent, low noise.”.

To demonstrate that the framework provides operationally meaningful explanations at decision time, we examine four trajectories drawn from the real-data test set representing the four qualitative outcomes an operator may encounter:

Case 1 – EDFA, true positive (traj 70).: OSNR declines steadily from 19 dB over 800 samples. The alarm fires at $t{=}724$, 44 s before the 15 dB crossing (Fig. 6). SHAP attribution at the alarm moment identifies current OSNR (−490 SHAP), rolling standard deviation (−85), SNR$_{t-1}$ (−55), and rolling mean (−45) as the dominant drivers–an operator-readable diagnostic of “current signal is low, recent history is consistently low with little noise, and this is a genuine degradation rather than a measurement transient.”

**Fig. 7:**
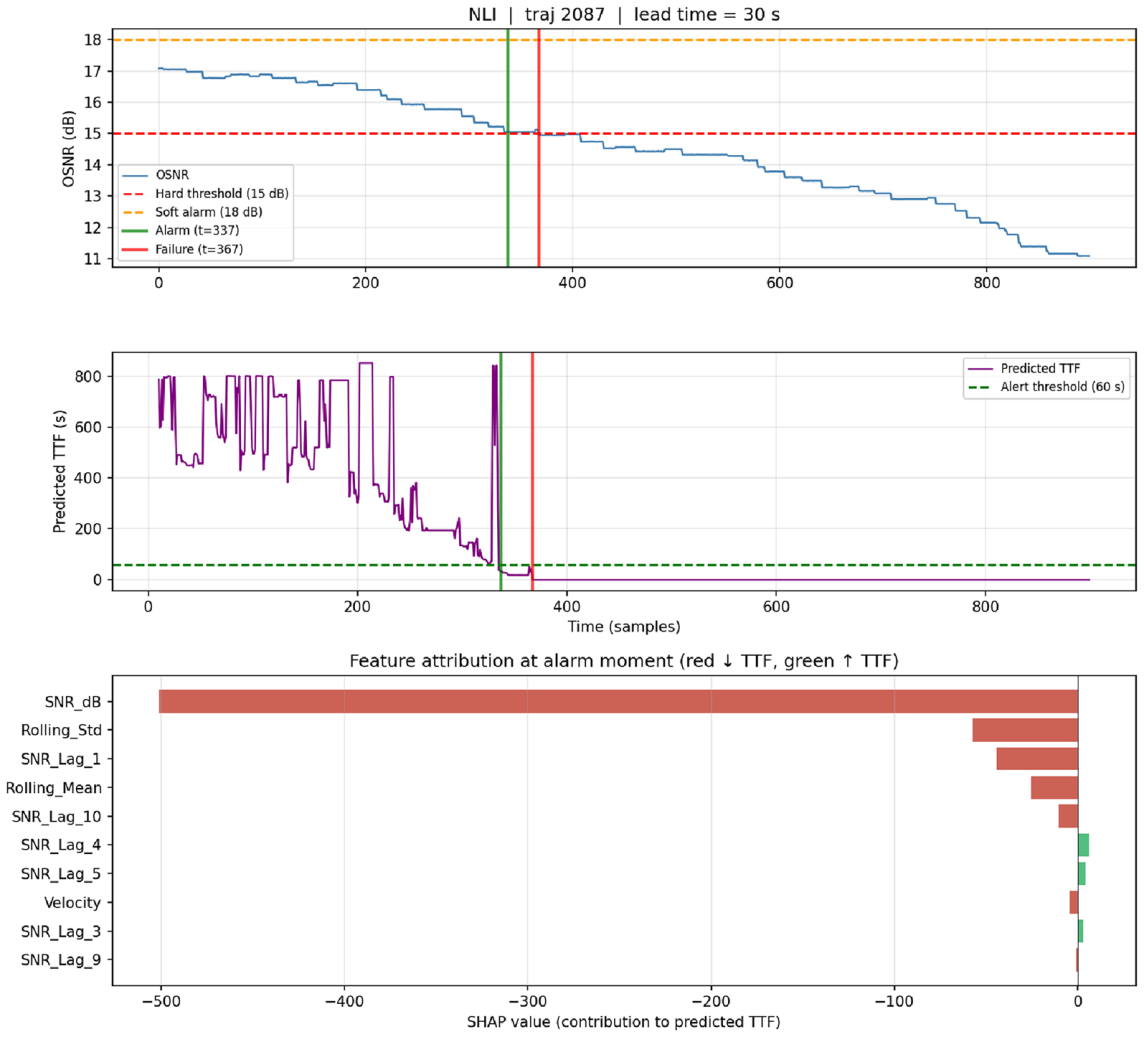
Case study 2 — NLI true-positive (trajectory 2087). Panel layout as in Fig. 6. Alarm at $t{=}337$, failure at $t{=}367$, lead time 30 s. Shorter lead reflects NLI’s accelerating dynamics. SHAP ranking at the alarm moment is consistent with the EDFA case, demonstrating that a single decision logic applies across physically distinct failure modes.

Case 2 – NLI, true positive (traj 2087).: OSNR declines sharply from 17 dB to 15 dB in 350 samples. The alarm fires at $t{=}337$, 30 s before failure (Fig. 7). The shorter lead time reflects the accelerating degradation characteristic of NLI. SHAP attribution at alarm follows the same ranking as the EDFA case, indicating that a single decision logic applies across physically distinct failure modes–a property valuable for operator training.

Case 3 – stable link, true negative (traj 1872).: OSNR remains at 19 dB for the full 900 samples. The predicted TTF remains pegged at the 880 s ceiling with small ($\sim$30 s) transient dips that never approach the 60 s alarm threshold. This demonstrates that the persistence filter and learned decision boundary combine to produce “trusted silence” on stable links.

**Fig. 8:**
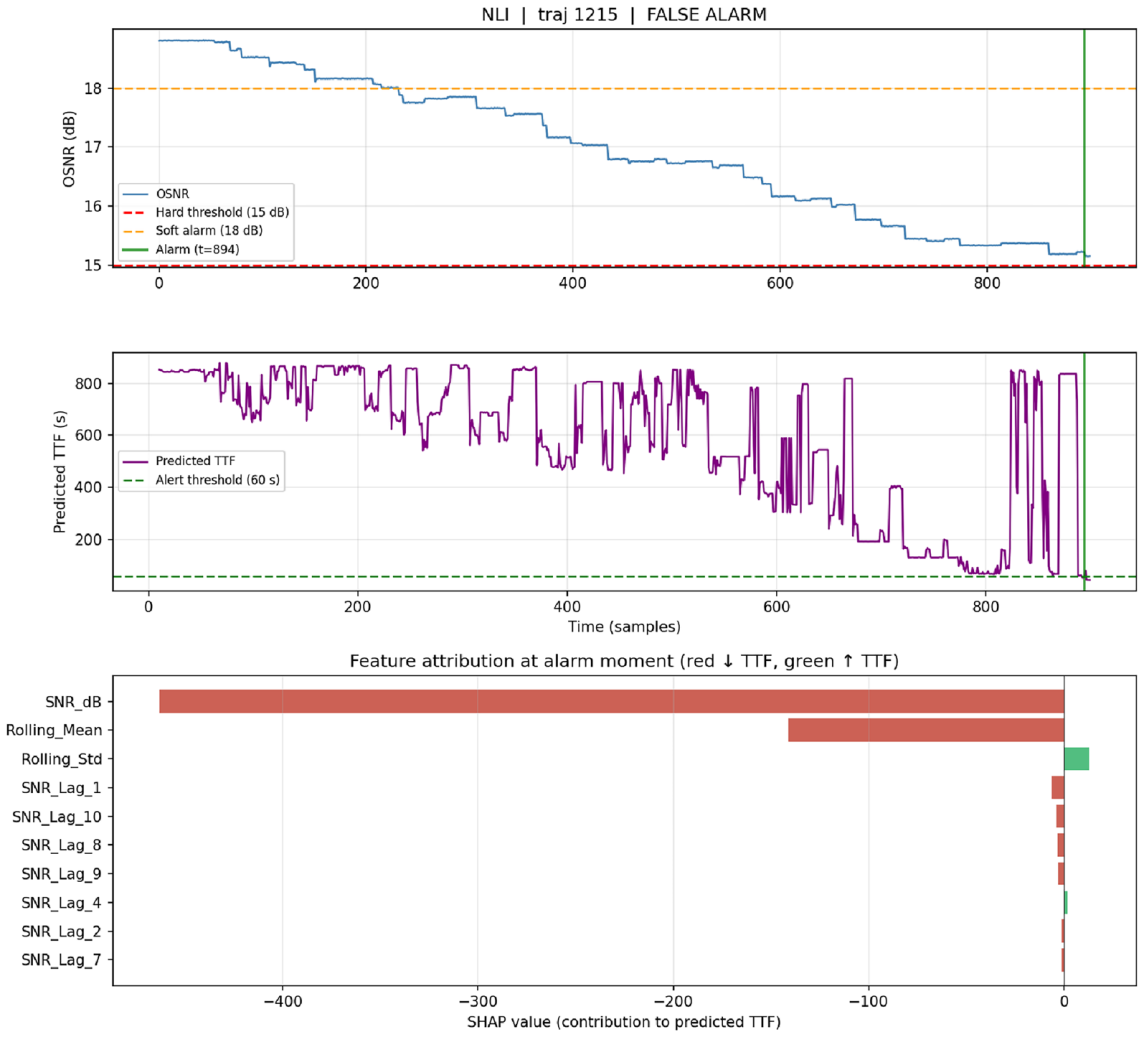
Case study 4 — false alarm (trajectory 1215). OSNR decays slowly from 18.8 dB toward $\sim$15.2 dB but does not cross the 15 dB threshold within the 900-sample observation window; alarm nonetheless fires at $t{=}894$. SHAP attribution shows the decision is driven by current OSNR and the rolling mean at marginal values—operator-interpretable reasoning that would likely resolve to a true positive had observation continued. Such marginal alerts illustrate the intended operational use of SHAP: support operator review and continuous learning.

Case 4 – false positive (traj 1215).: OSNR declines slowly from 18.8 dB to $\sim$15.2 dB over the full trajectory without crossing the 15 dB threshold within the observation window. An alarm fires at $t{=}894$, six samples before trajectory end (Fig. 8). SHAP attribution reveals that current OSNR and rolling mean drive the decision–reasoning that would likely resolve to a true positive had the observation continued beyond sample 900. This case illustrates the intended use of SHAP in production: operators can examine the explanation, classify marginal alerts, and feed disposition back for continuous learning.

### Global feature importance

**Fig. 9:**
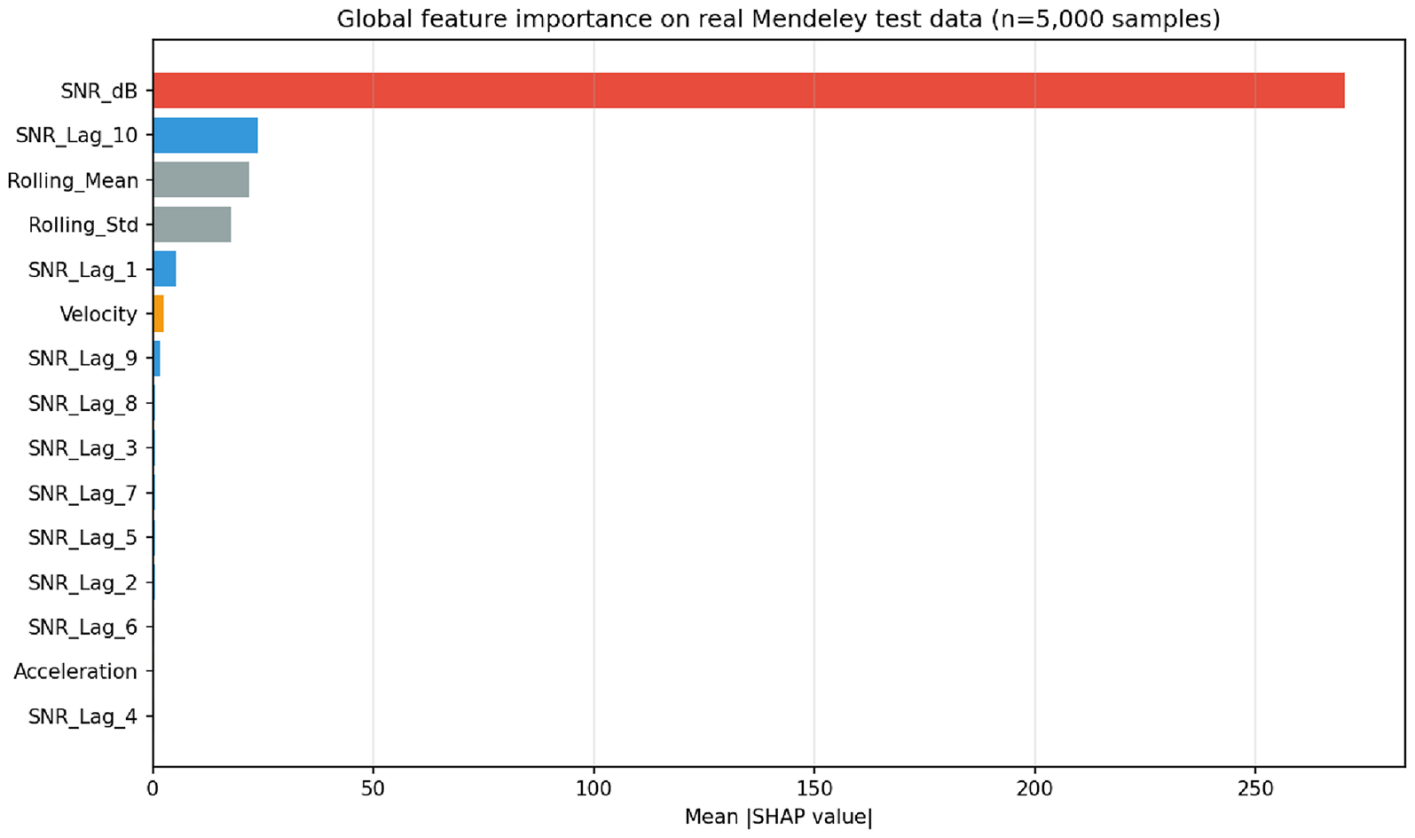
Global SHAP feature importance on 5,000 samples from the real Mendeley test set. Current OSNR contributes 77.9% of total mean-absolute SHAP; SNR$_{t-10}$ (6.9%), rolling mean (6.3%), and rolling standard deviation (5.1%) form the next tier. Derivative features contribute 0.1–0.75% in aggregate, but the per-alarm case studies (Figs. 6–8) show their contribution rises to 20–30% at decision moments.

Aggregating SHAP values across 5000 real test samples produces a markedly different distribution from the synthetic-data analysis(Fig. 9). On real data, current OSNR contributes 77.9% of total mean-absolute SHAP, with SNR$_{t-10}$ (6.9%), rolling mean (6.3%), and rolling standard deviation (5.1%) forming the next tier; velocity contributes 0.75% and acceleration 0.1%. This concentration reflects the smoother, less jittery character of real telemetry compared to stochastic-simulator trajectories (which inject controlled noise to differentiate failure modes).

*Interpretation:* at the aggregate level, current OSNR is the strongest single predictor. At alarm moments specifically (Sec. Per-Alarm Interpretability: Case Studies), rolling statistics contribute $\sim$25–30% of per-decision attribution. The derivative features remain informative for distinguishing among failure modes (as demonstrated in the cross-physics synthetic validation), but their contribution is concentrated at the decision boundary rather than uniformly across the operational envelope.

### End-to-end latency budget

**Fig. 10:**
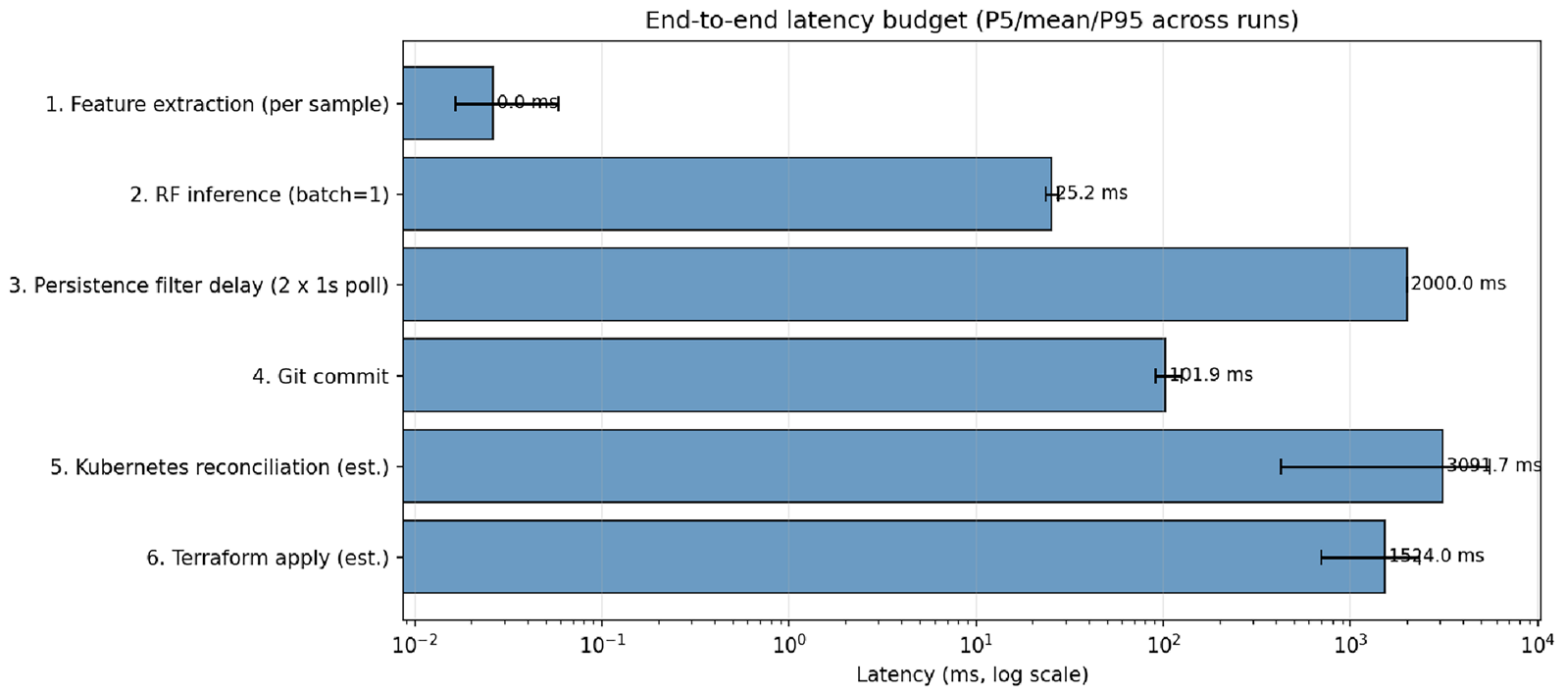
End-to-end latency budget of the proposed pipeline (log scale). Error bars indicate 5th/95th percentiles across 200 iterations per stage. ML-pipeline contributions (stages 1–2, totaling $\sim$25 ms) are below the readable range on the log axis; orchestration stages (5–6) dominate the budget, identifying them as the primary optimization target for future work.

Table 4 reports stage-wise wall-clock latency of the proposed pipeline, measured over 200 iterations per stage. Stages 1–4 are directly measured; stages 5–6 are estimated from published Kubernetes controller benchmarks and Sgambelluri et al.’s^16^ reported Terraform-to-OpenROADM apply times, as we do not currently operate a physical optical device in the loop (Fig. 10).

**Table 4:** Measured end-to-end latency budget (mean, milliseconds).

#	Stage	Mean (ms)	Source
1	Feature extraction	0.02	Measured
2	RF inference (batch=1)	25.2	Measured
3	Persistence filter (2$\times$1 s poll)	2000	Analytical
4	Git commit	101.9	Measured
5	Kubernetes reconciliation	3092	Estimated
6	Terraform apply	1524	Estimated^16^,
	Total (mean)	6743	

Three observations follow. First, *ML inference is negligible:* feature extraction plus RF inference totals 25.2 ms–less than 0.5% of the end-to-end budget. Physics-inspired tabular features plus tree inference yield a decision pipeline that is operationally invisible. Second, the persistence filter is the dominant *deliberate* delay, representing a design choice (two additional polls at 1 s intervals) that reduces the synthetic-data false-alarm rate from 12% to 2%. Third, orchestration stages (Kubernetes + Terraform) together account for 68% of the total budget. These stages are the legitimate optimization target for future work; options include pre-compiled Terraform plans, event-driven reconciliation instead of polling, and direct NETCONF APIs bypassing the Terraform layer for time-critical migrations.

The measured 6.7 s budget fits comfortably within the observed lead times from Sec. Per-Alarm Interpretability: Case Studies: 44 s for the EDFA case and 30 s for the NLI case. At the shortest NLI lead time, the pipeline consumes 22% of the available budget; at typical EDFA lead times, 15%. The orchestration layer is therefore fast enough to act within the prediction horizon for gradual failures. Although the real-data MAE of 73.2 s is larger than the simulation-only result (17.9 s), the shortest observed lead time (30 s for NLI failures) still exceeds the measured 6.7 s orchestration budget by 3.5$\times$, providing adequate operational margin for make-before-break migration.

## Discussion

### Scope and boundary conditions

The real-data characterization (Table 1) empirically establishes the scope of the method: OSNR-based prediction is effective for failures that produce a detectable OSNR signature (EDFA pump aging, NLI accelerating drift) and ineffective for failures that are AGC-compensated (ECL laser aging). On the Ghosh–Adhya benchmark, 52% of EDFA and 82% of NLI trajectories cross the 15 dB hard threshold in a 900-sample observation window; for ECL failures the figure is 0.5%–indistinguishable from the no-failure class.

This is not a limitation of the learning algorithm; it is a physical property of which failure modes are visible in the OSNR channel. Extension to ECL detection requires multi-channel feature fusion incorporating laser current (where ECL failures manifest as a +2 mA drift) and BER (where both EDFA and ECL produce signatures). Such extension is left to future work.

### Performance saturation and feature-engineering value

The < 1% MAE gap between Random Forest and XGBoost on the real benchmark, and the failure of higher-capacity deep models to improve on tree ensembles, together suggest that performance on this task is bounded not by model capacity but by the informativeness of the feature set. Physics-inspired derivative and rolling features capture most of the learnable signal; more expressive models incur additional variance (seed-to-seed instability, particularly for 1D-CNN) without lowering mean error. This is consistent with the tabular-data literature where well-engineered features plus tree ensembles remain competitive against deep sequence models^15^. It also argues against the alternative design choice–treating OSNR as a raw sequence input to a generic LSTM-which trades interpretability for no measurable accuracy gain.

### Relationship to physics-informed learning

Physics-Informed Neural Networks and related frameworks^13,14^ augment the *learning objective* with physical constraints (PDE residuals, conservation laws). Our approach augments the *feature space* with physics-inspired derivatives and statistics, retaining a conventional loss (squared error) and a conventional learner (Random Forest). We therefore describe the method as physics-*inspired* rather than physics-informed. The distinction matters because the two approaches make different claims: PINN-style methods claim that physics is enforced on the solution; feature-engineering methods like ours claim only that physics motivates the input representation. This is the more modest–and here, the demonstrably sufficient–claim.

### Operational latency and protection-switching context

The measured 6.7 s end-to-end latency is slower than classical 1+1 optical protection (50 ms per ITU-T G.873.1^27^) by two orders of magnitude. Our method is therefore not a replacement for fast protection switching–it complements it. Where 1+1 protection handles catastrophic events (fiber cuts, transceiver hard failures) with millisecond reaction, the proposed IaC pipeline addresses the preventable class of *gradual* soft failures that currently escalate to hard failures before any protection switch is triggered. The operational gain is measured not in switching time but in *avoided outages*: a successfully predicted soft failure never enters the protection-switch path.

### Energy feasibility estimate

We retain the energy-consumption analysis of the preliminary version as a feasibility estimate, not a validated measurement. The FEC-decoder power model used in the preliminary version, $P(S) = P_\text {static} + P_{\text {DSP,max}}\left[ (S_\text {ref}-S)/(S_\text {ref}-S_\text {crit})\right] ^\gamma$ with $\gamma \in [1.0, 2.0]$, uses literature-derived parameters from coherent transceiver datasheets and LDPC decoder theory^25,26^; the per-event Joule figures (54–77 J against an 18 dB reactive baseline) are consistent with the shape of soft-decision FEC power curves near the turbo cliff but have not been validated on commercial hardware. We flag this explicitly and do not claim energy savings as a primary benefit. The primary benefit is avoided service disruption; energy efficiency is a plausible secondary benefit pending hardware measurement.

### Limitations and future work

Beyond the ECL scope limitation already discussed, four limitations warrant acknowledgement. (1) The benchmark is time-compressed relative to real deployments (900-second degradation windows vs. days–weeks in the field); recalibration to operational timescales requires streaming field data. (2) The 756 lightpaths in the Mendeley benchmark, while public, derive from a single network simulator and may not capture carrier-specific physics (idiosyncratic fiber routes, particular amplifier vendors). (3) The Kubernetes and Terraform latency figures are literature estimates; on-hardware measurement in an operational NOC environment is needed. (4) Correlated multi-link failures (shared ducts, common equipment shelves) are outside the current model scope; graph neural networks or Bayesian propagation models are natural extensions.

## Conclusion

We presented a framework for proactive soft-failure prediction in optical transport networks, combining physics-inspired feature engineering, tree-ensemble learning, and Infrastructure-as-Code orchestration. The framework was validated on a controlled multi-physics simulation (17.9 s MAE, $R^2{=}0.914$) and on the Ghosh–Adhya (2025) real-data benchmark (73.2 ± 0.03 s MAE, $R^2{=}0.852$, $n{=}10$ seeds). Head-to-head comparison against XGBoost, LSTM, 1D-CNN, and three heuristic baselines under identical splits showed that tree ensembles with physics-inspired features achieve the lowest error, while <1% inter-estimator gap identifies the feature set as the dominant performance driver. Per-alarm SHAP attribution on four operational case studies–EDFA, NLI, stable-link, and false-alarm–demonstrated that the method produces operator-interpretable diagnostics at the decision moment. An end-to-end latency budget totaled 6.7 s, with ML inference contributing < 0.5%. The method is scoped to OSNR-visible gradual failures; extension to ECL and multi-link correlated failures constitutes the primary direction for future work.

## Data Availability

All data generated or analyzed during this study are included in this published article
